# Supplementary material for: Survival of Recombinant Monoclonal Antibodies (IgG, IgA and sIgA) Versus Naturally-Occurring Antibodies (IgG and sIgA/IgA) in an Ex Vivo Infant Digestion Model
Source: Nutrients. 2020 Feb 27;12(3):621. doi: 10.3390/nu12030621 (PMC7146391; doi:10.3390/nu12030621)
Supplement: Supplementary file 1 [file nutrients-12-00621-s001.zip › Supplementary Materials file 1.docx]

### **Supplementary Materials file 1**

### ***Palivizumab IgA and sIgA construction design, expression and purification***

### ***Construct design***

Constructs encoding antibody heavy and light chains were built from gBlock DNA fragments (IDT) and assembled using NEBuilder HiFi DNA Assembly Master Mix (NEB). Namely, the kappa-acceptor vector was built using a DNA fragment encoding a leader sequence from murine variable kappa chain (ATGGGTGTGCCCACTCAGGTCCTGGGGTTGCTGCTGCTGTGGCTTACAGATGCCAGATGC), separated by a NotI restriction site from the human kappa-chain constant region (IGKC*01) in the pcDNA3.4 (Invitrogen) backbone plasmid. Similarly, two alpha-chain acceptor vectors were built using a DNA fragment encoding a leader sequence from a murine variable heavy chain(ATGGAATGGAGCTGGGTCTTTCTCTTCTTCCTGTCAGTAACTACAGGTGTCCACAGC), separated by a NotI restriction site from the human alpha-chain constant regions (IGHA1*01 and IGHA2*01, each resulting in its own acceptor vector) in the pcDNA3.4 (Invitrogen) backbone plasmid. Additionally, a gamma-chain acceptor vector was generated using a DNA fragment encoding a leader sequence from a murine variable heavy chain, separated by a NotI restriction site from the human gamma-chain constant region (IGHG1*02) in the pcDNA3.4 (Invitrogen) backbone plasmid. Acceptor vectors were linearized by NotI (NEB) digestion and used in assembly with their respective variable-gene segments encoding Palivizumab (Synagis®) (GenBank: KC283077, KC283078) using NEBuilder HiFi DNA Assembly Master Mix (NEB), resulting in Palivizumab.hIgG1, Palivizumab.hIgA1, Palivizumab.hIgA2, Palivizumab.hIgK plasmids. A gBlock encoding human J-chain (hIgJ) sequence (GenBank: XM011531926) was assembled with the pcDNA3.4 (Invitrogen) backbone plasmid using NEBuilder HiFi DNA Assembly Master Mix (NEB). Human polymeric immunoglobulin receptor (hPIGR) construct was assembled from a cDNA clone (Sino Biological, cat. HG10131-UT), which was used to extract the fragment encoding the hPIGR ectodomain (amino acids 19–638), and place it between DNA fragments encoding tissue plasminogen activator leader peptide [1] and a C-terminal 8xHis tag followed by an AviTag [2] into the pcDNA3.4 backbone plasmid, resulting in the hPIGR.HisAvi plasmid.

### ***Protein expression and purification***

Protein production was performed using transient transfection of HEK293F cells, as previously described [3]. Briefly, recombinant hPIGR was produced by high-density PEI transfection with the hPIGR.HisAvi construct. Following 5 days of culturing, conditioned medium was harvested by centrifugation, and supplemented by the addition of NaN_3_ (0.02% final concentration) and NaCl (+350 mM, final concentration). Protein was then purified by immobilized metal-affinity chromatography using HisPur Ni-NTA resin (Thermo Scientific) followed by gel-filtration over a HiLoad 16/600 Superdex 200 pg column (GE Healthcare). For antibody production, HEK293F cells were co-transfected as follows: hIgG1/hIgK plasmids were used at 0.5/0.5 ratio, whereas hIgA1/hIgK/hIgJ or hIgA2/hIgK/hIgJ plasmids were used at 0.25/0.25/0.5 ratio. Following 5 days of culturing, conditioned medium was harvested by centrifugation, supplemented by the addition of NaN_3_ (0.02% final concentration), and NaCl (+350 mM, final concentration). Protein was then captured using Pierce Protein A Plus (Thermo Scientific), for IgG purification, or Pierce Protein L Plus Agarose (Thermo Scientific), for dIgA purification, washed with HBS-E-hs buffer (10 mM HEPES, pH 7, 300 mM NaCl, 2 mM EDTA), and eluted in 0.1 mM glycine, pH 2.7 (fractions were immediately pH-neutralized using 1 M Na_2_HPO_3_). Protein-containing fractions were pooled and buffer-exchanged against HBS-E (10 mM HEPES, pH 7, 150 mM NaCl, 2 mM EDTA) by ultrafiltration.

**References**

1. Wang, J.-Y.; Song, W.-T.; Li, Y.; Chen, W.-J.; Yang, D.; Zhong, G.-C.; Zhou, H.-Z.; Ren, C.-Y.; Yu, H.-T.; Ling, H. Improved expression of secretory and trimeric proteins in mammalian cells via the introduction of a new trimer motif and a mutant of the tPA signal sequence. *Appl. Microbiol. Biotechnol.* **2011**, *91*, 731-740, doi:10.1007/s00253-011-3297-0.

2. Fairhead, M.; Howarth, M. Site-specific biotinylation of purified proteins using BirA. *Methods Mol Biol* **2015**, *1266*, 171-184, doi:10.1007/978-1-4939-2272-7_12.

3. Carbonetti, S.; Oliver, B.G.; Vigdorovich, V.; Dambrauskas, N.; Sack, B.; Bergl, E.; Kappe, S.H.I.; Sather, D.N. A method for the isolation and characterization of functional murine monoclonal antibodies by single B cell cloning. *J. Immunol. Methods* **2017**, *448*, 66-73, doi:10.1016/j.jim.2017.05.010.
