# Supplementary material for: Survival of Recombinant Monoclonal Antibodies (IgG, IgA and sIgA) Versus Naturally-Occurring Antibodies (IgG and sIgA/IgA) in an Ex Vivo Infant Digestion Model
Source: Nutrients. 2020 Feb 27;12(3):621. doi: 10.3390/nu12030621 (PMC7146391; doi:10.3390/nu12030621)
Supplement: Supplementary file 1 [file nutrients-12-00621-s001.zip › Supplementary Materials file 2_3pm.docx]

### **Supplementary Materials file 2**

**Anti-idiotype palivizumab ELISA method**

The spectrophotometric ELISAs were recorded with a microplate reader (Spectramax M2, Molecular Devices, Sunnyvale, CA, USA) with three replicates of blanks, standards and samples. All ELISAs were performed according to the methods described by Bio-Rad with some modifications. Briefly, 100 μL per well of 1 μg/mL anti-palivizumab idiotype antibody HCA261 (Bio-Rad, Richmond, CA, USA) in 1x PBS was coated onto a clear flat-bottom Maxisorp 96-well plate (Nunc, Thermo Scientific, Waltham, MA, USA) and incubated overnight at 4°C. After incubation, the microtiter plate was washed three times with PBS with 0.05% Tween-20 (PBST) (Bio-Rad) and blocked for 1 h with 150 μL of PBST with 1% of bovine serum albumin (BSA) (Blocker^TM^ BSA (10x) in PBS, Thermo Scientific) at room temperature (RT). After washing three times, standards and samples were added to the wells (100 μL) and incubated for 1 h at RT. Standards were prepared using palivizumab in serial dilutions (from 0–1,000 ng/mL) in PBST with 1% BSA (Thermo Scientific). Fluid samples were diluted 200x and 400x (data were averaged) with PBST supplemented with 1% of BSA, added in wells (100 μL) and incubated at RT for 1 h. After incubating and washing, 0.16 μg/mL horseradish peroxidase (HRP)-conjugated goat anti-human IgG (Bio-Rad) was added to wells (100 μL) and incubated at RT for 1 h. After the plates were washed three times with PBST, 100 μL of the substrate 3,3’,5,5’-tetramethylbenzidine (1x, Invitrogen, San Diego, CA, USA) was added to the wells and incubated for 5 min at RT followed by addition of 50 μL of 2N sulfuric acid to stop the color reaction. Optical density was measured at 450 nm.
