# Supplementary material for: Survival of Recombinant Monoclonal Antibodies (IgG, IgA and sIgA) Versus Naturally-Occurring Antibodies (IgG and sIgA/IgA) in an Ex Vivo Infant Digestion Model
Source: Nutrients. 2020 Feb 27;12(3):621. doi: 10.3390/nu12030621 (PMC7146391; doi:10.3390/nu12030621)
Supplement: Supplementary file 1 [file nutrients-12-00621-s001.zip › Table S1.docx]

**Table S1.** Sequences of dimeric human IgA1, IgA2. Only the variable segment sequences were derived from palivizumab (Genbank: KC283077, KC283078). The sequences with variable-segment sequences were highlighted.

| **Formats** | **Sequence** | |
| --- | --- | --- |
| Palivizumab IgA1 | | **QVTLRESGPALVKPTQTLTLTCTFSGFSLSTSGMSVGWIRQPPGKALEWLADIWWDDKKDYNPSLKSRLTISKDTSKNQVVLKVTNMDPADTATYYCARSMITNWYFDVWGAGTTVTVSS**ASPTSPKVFPLSLCSTQPDGNVVIACLVQGFFPQEPLSVTWSESGQGVTARNFPPSQDASGDLYTTSSQLTLPATQCLAGKSVTCHVKHYTNPSQDVTVPCPVPSTPPTPSPSTPPTPSPSCCHPRLSLHRPALEDLLLGSEANLTCTLTGLRDASGVTFTWTPSSGKSAVQGPPERDLCGCYSVSSVLPGCAEPWNHGKTFTCTAAYPESKTPLTATLSKSGNTFRPEVHLLPPPSEELALNELVTLTCLARGFSPKDVLVRWLQGSQELPREKYLTWASRQEPSQGTTTFAVTSILRVAAEDWKKGDTFSCMVGHEALPLAFTQKTIDRLAGKPTHVNVSVVMAEVDGTCY |
| Palivizumab IgA2 | | **QVTLRESGPALVKPTQTLTLTCTFSGFSLSTSGMSVGWIRQPPGKALEWLADIWWDDKKDYNPSLKSRLTISKDTSKNQVVLKVTNMDPADTATYYCARSMITNWYFDVWGAGTTVTVSS**ASPTSPKVFPLSLDSTPQDGNVVVACLVQGFFPQEPLSVTWSESGQNVTARNFPPSQDASGDLYTTSSQLTLPATQCPDGKSVTCHVKHYTNPSQDVTVPCPVPPPPPCCHPRLSLHRPALEDLLLGSEANLTCTLTGLRDASGATFTWTPSSGKSAVQGPPERDLCGCYSVSSVLPGCAQPWNHGETFTCTAAHPELKTPLTANITKSGNTFRPEVHLLPPPSEELALNELVTLTCLARGFSPKDVLVRWLQGSQELPREKYLTWASRQEPSQGTTTFAVTSILRVAAEDWKKGDTFSCMVGHEALPLAFTQKTIDRLAGKPTHVNVSVVMAEVDGTCY |
