# Supplementary material for: Survival of Recombinant Monoclonal Antibodies (IgG, IgA and sIgA) Versus Naturally-Occurring Antibodies (IgG and sIgA/IgA) in an Ex Vivo Infant Digestion Model
Source: Nutrients. 2020 Feb 27;12(3):621. doi: 10.3390/nu12030621 (PMC7146391; doi:10.3390/nu12030621)
Supplement: Supplementary file 1 [file nutrients-12-00621-s001.zip › Table S2.docx]

**Table S2.** Average concentrations of palivizumab RSV F-protein specific IgG, IgA and sIgA from three ex vivo gastric and intestinal samples.

| **Samples** | **Average antibody concentrations in**  **ex vivo gastric contents (μg/mL) ^1^** | | **Average antibody concentrations in ex vivo intestinal contents (μg/mL) ^1^** | | |
| --- | --- | --- | --- | --- | --- |
|  | **0 h** | **1 h** | **0 h** | **1 h** | **2 h** |
| **Palivizumab IgG** | 51.73 | 41.76 | 66.10 | 52.85 | 42.50 |
| **Palivizumab IgA** | 125.66 | 104.37 | 150.89 | 133.30 | 119.83 |
| **Palivizumab sIgA** | 120.18 | 95.54 | 145.66 | 112.01 | 100.90 |

**^1^** Values are mean, *n* = 18.
