# Supplementary material for: Survival of Recombinant Monoclonal Antibodies (IgG, IgA and sIgA) Versus Naturally-Occurring Antibodies (IgG and sIgA/IgA) in an Ex Vivo Infant Digestion Model
Source: Nutrients. 2020 Feb 27;12(3):621. doi: 10.3390/nu12030621 (PMC7146391; doi:10.3390/nu12030621)
Supplement: Supplementary file 1 [file nutrients-12-00621-s001.zip › Table S3.docx]

**Table S3.** Average concentrations of naturally-occurring RSV F-protein specific IgG and sIgA/IgA from two ex vivo gastric and intestinal samples.

| **Samples** | **Average antibody concentrations in gastric samples (μg/ml) ^1^** | **Average antibody concentrations in intestinal samples (μg/ml) ^1^** |
| --- | --- | --- |
| **Naturally- occurring IgG** | 0.34 ± 0.11 | 0.33 ± 0.11 |
| **Naturally- occurring sIgA/IgA** | 3.41 ± 0.99 | 3.12 ± 0.84 |

**^1^** Values are mean ± SD, *n* = 12.
