# Supplementary material for: Survival of Recombinant Monoclonal Antibodies (IgG, IgA and sIgA) Versus Naturally-Occurring Antibodies (IgG and sIgA/IgA) in an Ex Vivo Infant Digestion Model
Source: Nutrients. 2020 Feb 27;12(3):621. doi: 10.3390/nu12030621 (PMC7146391; doi:10.3390/nu12030621)
Supplement: Supplementary file 1 [file nutrients-12-00621-s001.zip › Figure S1_v02202020_3pm.docx]

**Figure S1.** Effect of pH on stability of palivizumab spiked into human milk at 0 h and after 2 h of incubation at 37 °C with shaking at 300 rpm measured by anti-idiotype palivizumab ELISA. pH adjusted to 4, 7 and 8. Values are mean ± SD from 3 mother’s milk samples with 3 replicates at each of 2 dilutions. Two-way ANOVA followed by Tukey’s multiple comparisons test where means were compared between time 0 and 2 h for each sample.

**
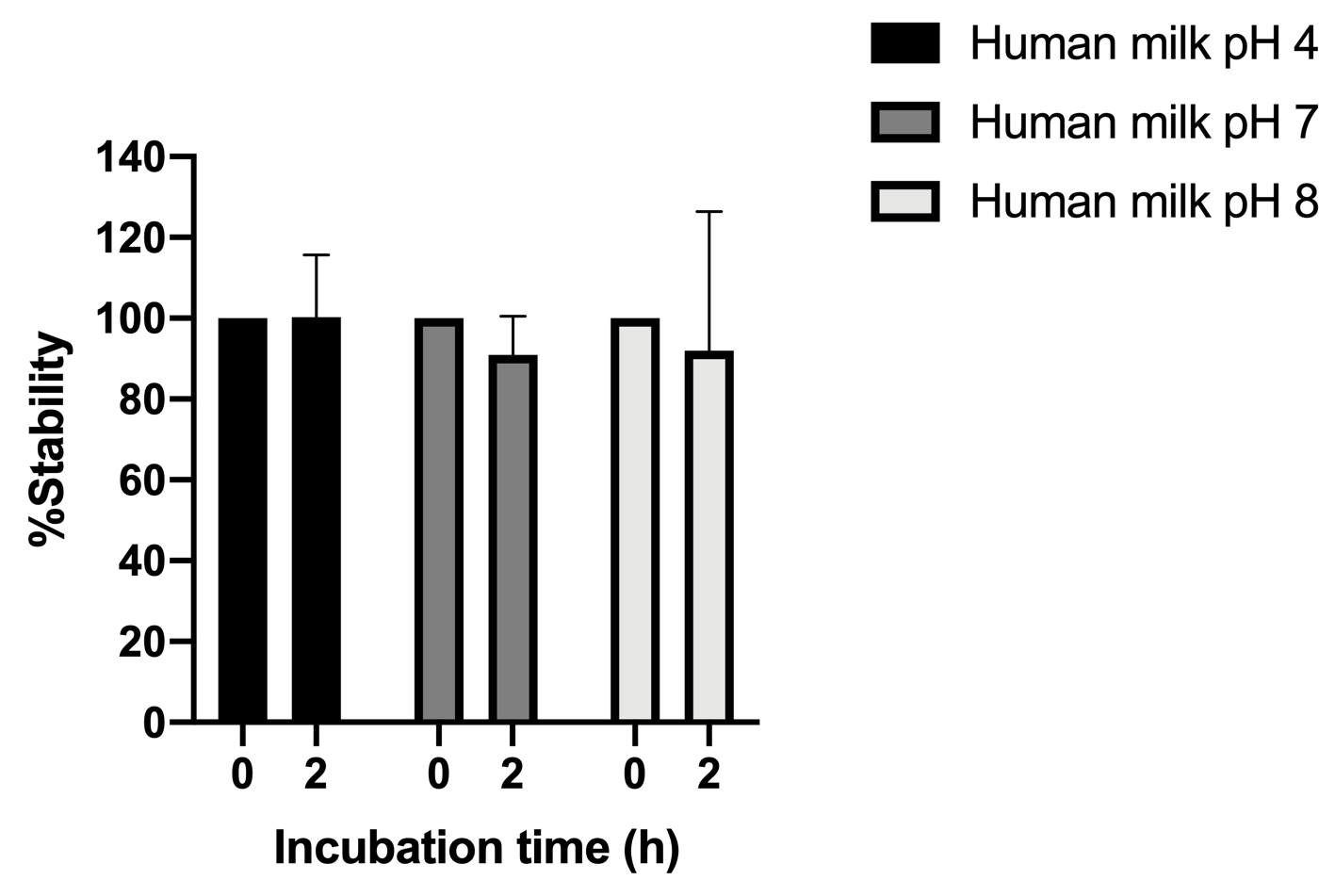
**
